# Incidence of Parkinson disease in North America

**DOI:** 10.1038/s41531-022-00410-y

**Published:** 2022-12-15

**Authors:** A. W. Willis, E. Roberts, J. C. Beck, B. Fiske, W. Ross, R. Savica, S. K. Van Den Eeden, C. M. Tanner, C. Marras, on behalf of the Parkinson’s Foundation P4 Group, Roy Alcalay, Michael Schwarzschild, Brad Racette, Honglei Chen, Tim Church, Bill Wilson, James M. Doria

**Affiliations:** 1 University of Pennsylvania, Philadelphia, PA USA; 2 Verge Genomics, San Francisco, CA USA; 3 Parkinson’s Foundation, Miami, FL USA; 4 Michael J. Fox Foundation, New York, NY USA; 5 Pacific Health Research and Education Institute, Honolulu, HI USA; 6 Mayo Clinic, Rochester, MN USA; 7 Kaiser Permanente Northern California, Oakland, CA USA; 8 University of California at San Francisco, San Francisco, CA USA; 9 University of Toronto, Toronto, CA USA

**Keywords:** Parkinson's disease, Movement disorders

## Abstract

Parkinson disease (PD) is the second most common age-related neurodegenerative condition diagnosed in North America. We recently demonstrated, using multiple epidemiological data sources, that the prevalence of PD diagnoses was greater than previously reported and currently used for clinical, research, and policy decision-making. Prior PD incidence estimates have varied, for unclear reasons. There is a need for improved estimates of PD incidence, not only for care delivery planning and future policy but also for increasing our understanding of disease risk. The objective of this study was thus to investigate the incidence of Parkinson disease across five epidemiological cohorts in North America in a common year, 2012. The cohorts contained data on 6.7 million person-years of adults ages 45 and older, and 9.3 million person-years of adults ages 65 and older. Our estimates of age-sex-adjusted incidence of PD ranged from 108 to 212 per 100,000 among persons ages 65 and older, and from 47 to 77 per 100,00 among persons ages 45 and older. PD incidence increased with age and was higher among males. We also found persistent spatial clustering of incident PD diagnoses in the U.S. PD incidence estimates varied across our data sources, in part due to case ascertainment and diagnosis methods, but also possibly due to the influence of population factors (prevalence of genetic risk factors or protective markers) and geographic location (exposure to environmental toxins). Understanding the source of these variations will be important for health care policy, research, and care planning.

## Introduction

Parkinson disease is a multi-system and multi-symptomatic neurodegenerative disorder, for which modifying or preventative measures are not presently available. As the population in Western nations has shifted to include a greater proportion of older adults, the public health and economic burdens of age-associated neurodegenerative disease have increased with an estimated economic cost of $52B per year in the US alone^1^. Indeed, the single greatest risk factor for PD is advanced age^2,3^.

Disease frequency in a given population can be measured as the annual prevalence (proportion of persons currently diagnosed with the disease) or incidence (proportion of persons newly diagnosed with the disease). Recently, we performed meta estimates of the 2010 prevalence of PD in North America, using multinational data from current and past epidemiology projects^4^. Our primary finding was that the overall prevalence of Parkinson disease among persons ages 45 and older was 572/100,000. We also found that PD burden in the population at ages 65 and above was higher than typically reported. These improved prevalence estimates and economic burden projections are but some of the vital statistics for population health. Prevalence is influenced not only by new cases appearing in the population but also by the survival of established cases. Incidence is an important complementary statistic in that it is a more direct reflection of the impact of risk factors for a disease (in the absence of changes in diagnostic efficiency). Improved estimates of disease incidence and mortality are also necessary for understanding disease risk, planning healthcare capacity, delivery, anticipating and addressing care disparities, and identifying unwarranted variations in care delivery.

Prior work has described the incidence of PD in limited populations such as isolated groups, cities, or lower population countries^5–10^. However, PD incidence data that are multinational or derived from multiple sources are not well documented. Health care systems, clinical registries, and clinical cohorts have the capability to detect and report PD burden. Although each data source contains varying degrees of patient representativeness, sensitivity, and specificity, the heterogeneity of the data underscores the need for disease burden estimates to include data from multiple patient sources. Therefore, building upon our efforts for improved estimates of PD prevalence, our group examined PD incidence across multiple regional cohorts and national datasets to generate an improved estimate of the incidence of Parkinson disease among older adults in North America. By aggregation of the available data, age and sex-stratified PD incidence estimates were derived. Additionally, the spatial clustering of Parkinson disease risk was explored.

## Results

### PD Incidence, ages 65+

Table 1 displays PD case and denominator numbers, incidence estimates for each dataset in this age group. PD incidence in persons ages 65 and older was examined over 9.3 million person-years. Study populations ranged in size from 138,806 (HAAS) to 6,866,623 (Medicare), and the number of incident PD cases identified ranged from 21 (REP) to 15,250 (Medicare).

**Table 1:** Incidence of Parkinson disease by study, sex, among adults ages 65 and older.

Study, sex, age	Population in person-years	Number of incident cases	Age-standardized rate (95% CI)^a^
*Both sexes 65+*			
Ontario	1,974,100	3660	185 (179–191)
Medicare	6,866,623	15,250	212 (208–215)
HAAS	–	–	–
KPNC	322,535	407	125 (113–138)
REP	19,579	21	108 (67–165)
*Males ages 65+*			
Ontario	876,387	2086	239 (229–250)
Medicare	2,628,112	7805	277 (270–283)
HAAS	138,806	185	128 (109–149)
KPNC	141,345	236	164 (143–186)
REP	8559	14	162 (88–272)
*Females ages 65+*			
Ontario	1,097,713	1574	143 (136–151)
Medicare	4,238,511	7445	161 (158–165)
HAAS	–	–	–
KPNC	181,190	171	95 (81–110)
REP	11,020	7	66 (27–136)

*HAAS* Honolulu-Asia Aging Study, *KPNC* Kaiser Permanente Northern California, *REP* Rochester Epidemiology Project.

^a^Standardized to the US 2010 Census population, based on 5-year age groups.

Age standardized PD incidence estimates for ages 65 and older ranged from 108 to 212 per 100, 000 person-years, from 162 to 277 among males, and from 66 to 161 among females. The Ontario Health care and Medicare program datasets produced incidence rates that were 1.5–2.0 times higher than found in among Kaiser Permanente Northern California members or the HAAS and Rochester Epidemiology Project cohorts.

### PD incidence, ages 45+

Table 2 displays PD incidence estimates for ages 45+, calculated using Ontario, KPNC and REP data. As expected, including low-risk age groups reduced overall PD incidence estimates substantially from 108 to 47/100, 000 (REP), from 125 to 53/100,000 (KPNC), and from 185 to 77/100,000 (Ontario).

**Table 2:** Incidence of Parkinson disease by study, sex, among adults ages 45 and older.

Study, sex, age	Population in person-years	Number of incident cases	Age-standardized rate (95% CI)^a^
*Both sexes 45+*			
Ontario	5,766,576	4577	77 (75–80)
KPNC	912,929	517	53 (48–57)
REP	58,252	28	47 (31–69)
*Males 45+*			
Ontario	2,752,896	2637	93 (90–97)
KPNC	421,478	308	66 (59–74)
REP	26,884	18	64 (38–102)
*Females 45+*			
Ontario	3,013,680	1940	63 (61–66)
KPNC	491,451	209	40 (35–46)
REP	31,368	10	33 (16–60)

*KPNC* Kaiser Permanente Northern California, *REP* Rochester Epidemiology Project.

^a^Standardized to the US 2010 Census population, based on 5-year age groups.

### Age trends in PD Incidence

As examined in persons aged 65 and older, PD incidence estimates increased with age in the decades 65–74 years, 75–84 years in every study sample. However, at 85+ years, divergent incidence trends were found across datasets. Specialist confirmed cases in the HAAS, KPNC, and REP declined slightly from the previous decade (from 199 to 139 per 100,000, from 222 to 165 per 100, 000 and from 216 to 198 per 100,000 respectively), whereas the two administrative datasets (Ontario and Medicare) estimated that the incidence of PD diagnosis with concurrent anti-PD medication use continued to increase.

### Sex differences in PD incidence

**Fig. 1:**
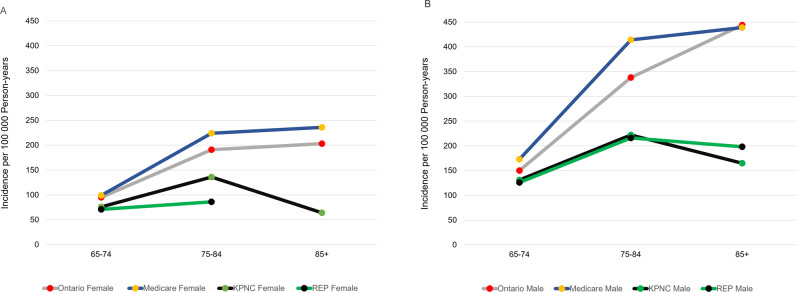
Age- and Sex-Specific Incidence Rates of PD. Age-Specific Incidence Rates per 100 000 Person-years for (panel **A**) Females and (panel **B**) Males of: Treated Parkinsonism in the Ontario population (1,974,100 person-years, 2012); Treated Parkinsonism in the U.S. Medicare Insured population (6,866,623 person-years, 2012); Treated, Neurologist Reviewed Parkinson Disease in the Kaiser Permanente Northern California (KPNC) Insured Population (322,535 person-years, 2012); Movement Disorders Specialist Consensus Parkinson Disease in the Honolulu Asian Aging Study (HAAS) Cohort (138,806 person-years, 2011).

As shown in Fig. 1, incidence estimates were higher in males as compared to females at all ages in the datasets which allowed sex-based comparisons into the 8th decade (Ontario, Medicare and KPNC; no incident cases were reported for females ages 85+ in the REP). PD incidence among males rose first, most sharply between ages 64 and 74 in the fourstudy cohorts. In the decade of peak incidence, the male: female rate ratio varied across datasets: 1.86 (Medicare, 85+ years), 2.18 (Ontario 85+ years), 2.51 (REP, 75-84 years) 2.58 (KPNC, 75–84 years). When individuals below the age of 65 were included, a higher incidence of PD among males remained; however, the male: female sex ratio declined −4.6% (KPNC), −11.4% (Ontario), −20.8% (REP) (Table 3).

**Table 3:** Male to female ratio, age-standardized PD incidence, 2012.

Study	Sex rate ratio (Male: Female)		
	Ages 65+	Ages 45+	Percent difference (Δ%)
Ontario	1.66 (1.55, 1.77)	1.49 (1.40, 1.58)	−10.8 (10.1, 11.3)
Medicare	1.69 (1.64, 1.74)	–	–
KPNC	1.77 (1.45, 2.15)	1.71 (1.44, 2.05)	−3.4 (0.7, 4.7)
REP	2.57 (1.04, 6.38)	2.10 (0.97, 4.55)	−20.1 (6.9, 33.6)

*KPNC* Kaiser Permanente Northern California, *REP* Rochester Epidemiology Project.

### Geographical variation in PD incidence

**Fig. 2:**
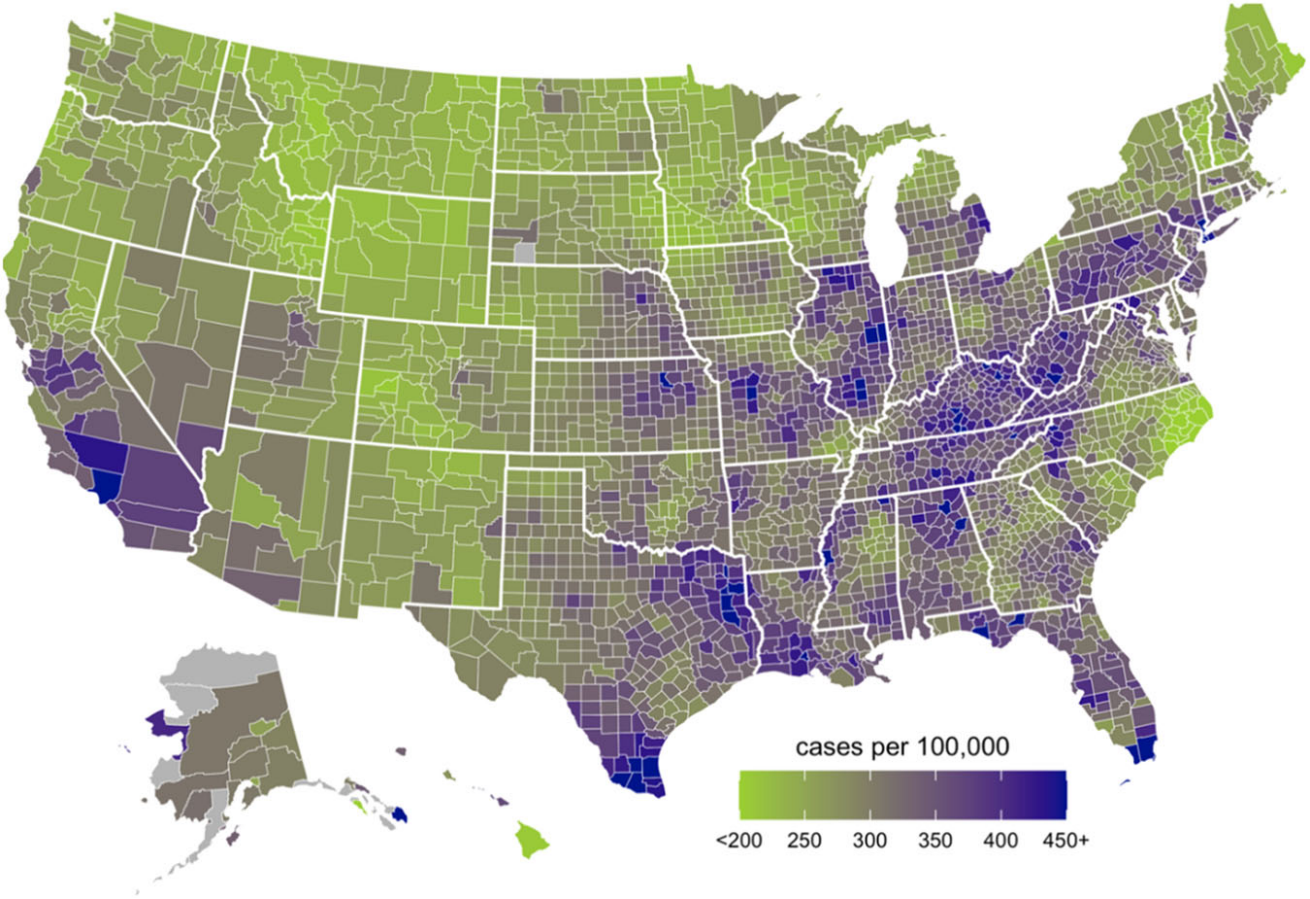
Geographical variation in 2012 PD Incidence among 6,866,623 Medicare beneficiaries.

The age and sex adjusted county rates of newly diagnosed, treated Parkinson disease among eligible Medicare beneficiaries, with Bayesian hierarchical modeling and smoothing to increase the precision of estimates in less populous areas, is shown in Fig. 2. A clustering of counties with a higher incidence of PD was observed at the juxtaposition of the Midwestern and Southern regions of the United States. Other higher incidence areas were found in southern California, southeastern Texas, central Pennsylvania, and Florida. Lower incidence areas included the Mountain West region, the western Midwest, and the far Northwest.

## Discussion

Parkinson disease is the second most common age-related neurodegenerative disease in the world and is responsible for significant disability and increased risks of dementia and mortality^11–14^. Data drawn from younger onset cases, multi-incident families, individuals with risk markers for PD are crucial for exploring the molecular mechanisms of disease and identifying the clinical and biological changes occurring in the preclinical and prodromal periods, which may ultimately lead to effective neuroprotection^15–26^. In turn, population-level estimates of PD burden are crucial for health care and health policy, for advocating for appropriate research funding and insurance coverage for treatment and for minimizing drug shortages. PD incidence estimates may be influenced by multiple factors, including population age, geographical location, the prevalences of genetic and environmental risk and protective markers, and case ascertainment and diagnosis methods. Comparisons of incidence estimates from multiple datasets, using different case ascertainment and diagnosis methods, can demonstrate these variations. In this study, we report incident rates of Parkinson disease in North America, measured simultaneously across five PD epidemiology data sources which sampled middle age and older adult populations. We found that Parkinson disease incidence in persons ages 65 and older ranged from 108 to 212 per 100,000 persons. Among persons aged 45 and older, PD incidence ranged from 47 to 77 per 100,000 persons. As expected from prior studies^5^, PD incidence increased with age and was higher among males. We also found the U.S. “PD belt”, a clustering of PD diagnoses originally described using PD incidence estimates from 2005^27^, was still present in 2012.

The highest PD estimates were generated from the diagnostic algorithm applied to the Ontario and Medicare administrative datasets. This algorithm captured cases of parkinsonism, categorized as Parkinson disease, for which the person was prescribed at least one motor symptom medication. Another study of administrative claims, from the French National Health Insurance system (*Système National d*’*Information Inter-Régimes de l*’*Assurance Maladie*) found comparable incidence rates, ranging from 74 to 140 (ages 65–74), 210–277 (ages 75–84) and 299 (ages 85–89) per 100,000^28^. Higher PD incidence estimates are not limited to studies which use administrative claims data and broad ascertainment methods. In a prospective cohort study of male physicians, PD incidence ranged from 134/100,000 person-years (among participants ages 65–69) to 614.7/100,000 person-years among participants 85–89 years old^9^.

The HAAS, REP, and KPNC studies, which produced lower incidence estimates, required high levels of clinical evidence and diagnosis by an expert clinician (a neurologist or movement disorders neurologist), even after screening, similar to the diagnostic protocols used for entry into a PD research study or clinical trial. Using this approach, fewer potential incident PD diagnoses would be expected to have all required diagnostic criteria documented in the medical record. Additionally, individuals who had infrequent visits, providers from multiple systems, low-quality interactions with health care providers, or had confounding comorbidities (such as psychiatric conditions, prior stroke, neuropathy, multifactorial gait disorders) would be expected to more frequently have inadequate documentation for meeting study diagnostic criteria in a retrospective analysis of real-world clinical practice.

The lower incidence rates captured in the HAAS, REP, and KPNC cohorts may also be influenced by actual differences in PD risk factors experienced by the populations in these diverse geographic areas. Compared to the general Medicare population, Medicare-aged adults who purchase KPNC insurance have higher income and higher rates of college education^29^. These social determinants of health associate with higher quality health care and with exercise^30,31^. Exercise is a health behavior which may protect against developing PD and has been demonstrated to preserve function after diagnosis^32–35^. Exercise is also associated with a lower risk of diabetes^36,37^, which several studies have suggested is a risk marker for PD or PD progression^38–42^. The HAAS is based on a cohort of Japanese- American men. Epidemiological data have reported lower PD incidence in Asian countries or among persons identified as Asian^27,43^. Potential reasons for the lower risk in Asians include lower prevalence of pathogenic genetic risk factors (e.g., LRRK2 or GBA mutations), differential exposure to risk, or resilience to PD-like neurodegeneration due to lifestyle or diet^44,45^. Our PD map suggests that KPNC, REP and HAAS participants live in regions of the U.S. with relatively lower incidence rates, which may also reflect regional differences in environmental exposures.

The primary risk factor for PD is age^2^, and within all cohorts, we found that PD incidence increased into the 7th decade. Thereafter, two distinct age trends emerged. For the KPNC, REP, and HAAS data, incidence rates fell slightly, whereas for the Ontario and Medicare data studies, PD incidence continued to increase. These two patterns in age-stratified PD incidence have been found in multiple other epidemiological studies^6,9,10,26,27^. The widening of the calculated confidence intervals at higher age strata limits the ability to interpret these findings, but one potential explanation for the relatively lower number of diagnoses in the KPNC, REP, and HAAS datasets is that it may become more difficult to identify mild parkinsonism in the oldest adults, particularly those with multiple comorbidities, and to differentiate early PD from normal advanced aging. As a result, lower ascertainment would be expected. The Ontario and Medicare criteria for PD were not subject to the application of specific PD diagnostic criteria but rather relied on the treating physician’s judgment and coding of the most likely diagnosis. To ensure those with PD are diagnosed early, brief tools for parkinsonism screening in routine primary care clinical practice, such as those for dementia^46^, are needed. This is especially true given the evidence that early diagnosis of parkinsonism secondary to PD associates with improved patient outcomes and clinician decision making^47–51^.

Symptomatic cognitive dysfunction or dementia, due to Alzheimer disease, vascular dementia, toxic or metabolic cognitive dysfunction, increases with age^52^ and becomes very prevalent in the 8^th^ decade and beyond^12,53,54^. Although mild cognitive dysfunction at the time of PD diagnosis is common^55^, PD diagnostic criteria used in practice and in most of our studies required there to be no evidence of moderate or severe cognitive impairment at the time of or soon after motor symptom onset. The strict application of this exclusion criterion would favor underdiagnosis of PD in subpopulations at higher risk for concomitant cognitive dysfunction/dementia, most noticeably individuals in the 8th decade and beyond. PD patients from higher risk groups (due to genetic factors or occupational exposures) have been observed to present as early onset cases, another phenomenon that favors lower observed incidence with advancing age^56–60^. Epidemiological studies which include the use of valid PD biomarkers, when available, may clarify the true incidence of PD in the highest age groups, as well as within patient subgroups that have a high prevalence of primary or secondary cognitive impairment (e.g., those with cerebrovascular disease, renal dysfunction).

The male: female ratio for incident PD increased with age in all epidemiological cohorts in our study, consistent with the previous data that supports the hypothesis that biological sex is an intrinsic risk factor for PD^61–65^. A 2015 meta-analysis of PD incidence data from 22 studies which included age/sex-specific data found the overall M:F incidence ratio was 1.57 (95% CI 1.46 to 1.68, *p* < 0.001), and that age-specific pooled M:F incidence ratios increased from 1.46 (95% CI 1.33–1.61) between 60 and 79 years to 1.93 (95% CI 1.84–2.03) in persons above 80 years^28^. Our finding of a decrease in the M:F sex ratio when we included individuals with age at onset as low as 45 indirectly supports the theory that sex differences in PD risk increase with age. Monogenic risk factors for PD are equally distributed in males and females^66^, and associate with earlier onset disease. Sporadic PD risk is positively correlated with age, as are the potential cumulative influences of non-genetic risk markers that may differ by gender, including occupational, neuroendocrine, and hormonal exposures, lifestyle factors^67–69^.

In this study, we estimated the incidence of Parkinson disease simultaneously across five epidemiological datasets using commonly applied diagnosis and case ascertainment methods applied to 2012 data. We established a range of total incident PD diagnoses in North America of approximately 60,000 to 95,000 among adults ages 45 and older. Using the Medicare administrative database alone for this same time period suggests an incident rate of PD of nearly 90,000 per annum just for those 65 and older. As discussed above, regarding the ascertainment methods for each dataset, this range in incidence likely represents the lower and upper bounds of those diagnosed with PD in 2012. Nevertheless, this contrasts sharply with the estimate of 40,000-60,000 new cases per annum that has been cited previously for PD incidence^6,10^. The reasons for a greater incidence as compared to prior studies, particularly those drawn from data prior to 2010 ^6,10,70^, remains to be explained, but could represent either improved ascertainment and clinical recognition of PD or reflect the impact of risk factors for PD. The confirmation of an increased trend of PD in different geographic areas, populations, and datasets does not support the theory that our findings are spurious.

Because of the significant implications for health care policy, research, and care planning, we propose a working estimate of a PD incident rate of 62 per 100,000 person-years for those 45 and older. For 2012 population data, this rate would equate to 77,000 diagnosed PD cases per annum; for 2020, the number of diagnosed PD cases rises to 86,000 per annum assuming negligible changes in age distribution from our standardization year (2010). The growth in those diagnosed and living with PD underscores the need for policy makers to confront an increasing strain on clinical services^71–74^ as well as the need to provide additional funding for research that can lead to improved therapies if not an outright cure.

Our study, like all retrospective studies, has several limitations. It is prone to measurement error that can be due to confounding, misclassification, miscoding and selection bias, among others. Our estimates were primarily derived using data from completed epidemiological studies, limiting our ability to apply new or varying diagnostic and ascertainment criteria across datasets. PD incidence rates for the current year may be even higher due to decreased prevalence of alleged protective factors (e.g., smoking), increased prevalence of risk markers^26^ (e.g., diabetes, cognitive dysfunction, lack of physical activity, pesticides)^26,75^, or greater clinical recognition of PD symptoms, particularly among older adults with comorbidities. In contrast, PD incidence trends from 2012 to now may be negatively affected by reduced community and occupational exposure to environmental toxicants^10^ or lower case ascertainment due to COVID-related changes in health care-seeking behavior.

Our study highlights the need for more work throughout the translational epidemiology continuum and the potential benefits of harmonizing case ascertainment and diagnostic criteria across data sources. Obtaining high-quality incidence rates in multiple populations is fundamental to understanding what is needed to address the burden of the disease and to plan for adequate healthcare services. Although population-based PD registries would be ideal and have been tried in some areas^75–78^, setting up and maintaining a disease registry has proven to to be logistically difficult and expensive. Alternative approaches and data sources, such as those included here, can provide high-quality estimates for both the prevalence and incidence of disorders such as Parkinson’s disease.

## Methods

### Ethical approval/human study research protection

The work was approved by the following research ethics committees:

HAAS: Kuakini Medical Center, Honolulu, HI, and Veterans Affairs Pacific Islands Health Care System, Honolulu, HI Institutional Review Boards.

KPNC: KPNC Institutional Review Board, Oakland, CA

Medicare: The Human Subjects Research Protection Program, the University of Pennsylvania.

Ontario, Canada: The Research Board at Sunnybrook Health Sciences Centre, Toronto.

REP: The Institutional Review Boards of Mayo Clinic and Olmsted Medical Center, Rochester, MN.

All datasets provided for analysis in this study were fully deidentified, data were presented for analysis and shared only in aggregate form. The requirement for written participant consent was waived by each ethics research committee for this study.

#### Data sources

Parkinson’s disease incidence in the year 2012 was calculated using data from five data sources in North America: (1) Kaiser Permanente Northern California (KPNC), (2) the Honolulu-Asia Aging Study (HAAS), the (3) Rochester Epidemiology Project (REP), (4) the U.S. Medicare program and (5) the Ontario health administrative databases (Ontario). Individual project descriptions and ascertainment methods are detailed in Supplemental Table 1.

#### PD Ascertainment

The ascertainment and diagnostic criteria for incident PD were applied per original project criteria and generally consisted of new documentation of PD symptoms or a PD diagnosis in the year 2012, as of July 1, which was defined as the incidence day. The HAAS cohort did not have data in 2012, therefore incidence data were drawn from 1965-2011. As shown in Table 4, ascertainment and diagnostic criteria varied between datasets. In the HAAS cohort, potential PD cases were primarily identified through regular in-person screening exams, followed by a standardized research physical examination by a neurologist. The final diagnosis was made by consensus of movement disorders experts after a case review. Incident PD cases in the KPNC dataset were identified via medical record examination using an algorithm requiring multiple PD diagnoses, antiparkinson treatment and procedures, and considered counterfactual information and the diagnosing physician specialty (e.g., non-neurologist, neurologist, movement disorder specialist). The REP began with a similar medical record screening approach, which was then followed by a neurologist review of potential cases for documentation of additional qualifying/disqualifying features.

**Table 4:** Study populations and case ascertainment methods.

Study (Location)	HAAS (Hawaii, USA)	KPNC (California, USA)	REP (Minnesota, USA)	Ontario Health Care (Ontario, Canada)	Medicare program (USA)
Study population	Longitudinal Honolulu Heart Program cohort study participants: 8006 Japanese American men born between 1900 and 1919. Study cohort was assembled in Honolulu in 1965. Surveillance through 2011.	Participating members of a closed, integrated health care delivery system, Kaiser Permanente North California.	Residents of Olmsted County, Minnesota, USA	Residents of Ontario Canada, receiving health care from the provincial government.	Residents of the United States ages 65 and above, receiving health care from the federal government
Study data components, sources for PD case ascertainment	Longitudinal cohort data (screening and confirmatory examinations), medical records.	Medical records for care delivered in the inpatient outpatient settings, plus prescribing data.	Electronic medical records	Health care administrative claims for care delivered in the inpatient and outpatient settings, prescription claims.	Health care administrative claims for care delivered in the inpatient and outpatient settings, prescription claims.
PD diagnostic criteria	Movement disorders neurologist consensus diagnosis, made after review of research and medical records, qualifying research clinical examination.^1^	Algorithm that includes multiple PD diagnoses by a qualified physician, PD motor symptom medication.	Neurologist diagnosis, made after medical records review of potential cases identified via electronic screen^2^	Algorithm that includes two outpatient or one inpatient PD diagnosis (ICD-9 code = 332 or ICD-10 code = G20), made by a physician or advanced practice provider, plus at least one antiparkinson medication prescription fill.^a^	Algorithm that includes two outpatient or one inpatient PD diagnosis (ICD-9 code = 332 or ICD-10 code = G20), made by a physician or advanced practice provider, plus at least one antiparkinson medication prescription fill.

*PD* Parkinson Disease, *ICD* International Classification of Disease, *HAAS* Honolulu Asia Aging study, *KPNC* Kaiser Permanente Northern California, *REP* Rochester Epidemiology Project

^a^Medication dispensing data available only for those over age 65.

Notably, the same case ascertainment algorithm was applied to the two administrative claims databases representing the Ontario Healthcare and U.S. Medicare programs for this study. Incident PD was identified as two physician or advanced practice provider diagnosis claims made in the outpatient setting, at least 30 days apart, or, one inpatient PD diagnosis, among individuals receiving program benefits for at least two years without prior diagnosis. Additionally, potential cases must have had at least one prescription fill for an antiparkinson disease medication within six months of the first diagnosis.

### Statistical analysis

We calculated age-sex-standardized incidence rates^79^ of Parkinson disease among persons ages 65 and above for each dataset. We repeated these analyses for individuals ages 45 and above, using the three datasets (Ontario, KPNC, REP) which contained routinely captured information on persons below age 65. Sex-specific incidence rates were calculated, standardized to the U.S. 2010 population using 5-year age strata^80^. Race and ethnicity were defined inconsistently across datasets; therefore, we made no attempts to produce race/ethnicity-specific incidence rates. Taking advantage of the fact that the U.S. Medicare program has insured members in all states, we used this dataset to build a spatial hierarchical Bayesian model^81^ of county-level, age- and sex-adjusted PD incidence and produce a map display of the spatial variation in incident PD.

### Reporting summary

Further information on research design is available in the Nature Research Reporting Summary linked to this article.

## Supplementary information

Supplemental Table 1

Reporting Summary

## Data Availability

Due to data-sharing, data-use or privacy agreements, data cannot be made available. Analytic code can be made available upon request to the corresponding author.

## References

[1] Yang W (2020). Current and projected future economic burden of Parkinson’s disease in the U.S. NPJ Parkinsons Dis..

[2] Reeve A, Simcox E, Turnbull D (2014). Ageing and Parkinson’s disease: why is advancing age the biggest risk factor?. Ageing Res Rev..

[3] de Lau LM, Breteler MM (2006). Epidemiology of Parkinson’s disease. Lancet Neurol..

[4] Marras C (2018). Prevalence of Parkinson’s disease across North America. NPJ Parkinsons Dis..

[5] Hirsch L, Jette N, Frolkis A, Steeves T, Pringsheim T (2016). The incidence of Parkinson’s disease: a systematic review and meta-analysis. Neuroepidemiology.

[6] Van Den Eeden SK (2003). Incidence of Parkinson’s disease: variation by age, gender, and race/ethnicity. Am. J. Epidemiol..

[7] Savica R, Grossardt BR, Bower JH, Ahlskog JE, Rocca WA (2013). Incidence and pathology of synucleinopathies and tauopathies related to parkinsonism. JAMA Neurol..

[8] Bower JH, Maraganore DM, McDonnell SK, Rocca WA (1999). Incidence and distribution of parkinsonism in Olmsted County, Minnesota, 1976–1990. Neurology.

[9] Driver JA, Logroscino G, Gaziano JM, Kurth T (2009). Incidence and remaining lifetime risk of Parkinson disease in advanced age. Neurology.

[10] Savica R, Grossardt BR, Bower JH, Ahlskog JE, Rocca WA (2016). Time trends in the incidence of Parkinson disease. JAMA Neurol..

[11] Dorsey E, Sherer T, Okun MS, Bloem BR (2018). The emerging evidence of the Parkinson pandemic. J. Parkinson’s Dis..

[12] Gardner RC, Valcour V, Yaffe K (2013). Dementia in the oldest old: a multi-factorial and growing public health issue. Alzheimer’s Res. Ther..

[13] Murray CJ (2012). Disability-adjusted life years (DALYs) for 291 diseases and injuries in 21 regions, 1990–2010: a systematic analysis for the Global Burden of Disease Study 2010. lancet.

[14] Willis AW (2012). Predictors of survival in patients with Parkinson disease. Arch. Neurol..

[15] Biddiscombe KJ, Ong B, Kalinowski P, Pike KE (2020). Physical activity and cognition in young-onset Parkinson’s disease. Acta Neurol. Scand..

[16] Ishiguro M (2021). Genetic analysis of ATP10B for Parkinson’s disease in Japan. Parkinsonism Relat. Disord..

[17] Kaiyrzhanov R (2021). A glimpse of the genetics of young-onset Parkinson’s disease in Central Asia. Mol. Genet. Genom. Med..

[18] Kukkle PL (2021). Clinical study of 668 indian subjects with juvenile, young, and early onset Parkinson’s Disease. Can. J. Neurol. Sci..

[19] Mehanna R, Jankovic J (2019). Young-onset Parkinson’s disease: Its unique features and their impact on quality of life. Parkinsonism Relat. Disord..

[20] Post B (2020). Young onset Parkinson’s disease: a modern and tailored approach. J. Parkinsons Dis..

[21] Schapira AHV, Morris HR (2020). Pathogenetic insights into young-onset Parkinson disease. Nat. Rev. Neurol..

[22] Schirinzi T (2020). Young-onset and late-onset Parkinson’s disease exhibit a different profile of fluid biomarkers and clinical features. Neurobiol. Aging.

[23] Gialluisi A (2021). Identification of sixteen novel candidate genes for late onset Parkinson’s disease. Mol. Neurodegener..

[24] Wang Y (2021). LRRK2-NFATc2 pathway associated with neuroinflammation may be a potential therapeutic target for Parkinson’s disease. J. Inflamm. Res..

[25] Foo JN (2020). Identification of risk loci for Parkinson disease in Asians and comparison of risk between Asians and Europeans: a genome-wide association study. JAMA Neurol..

[26] Heinzel S (2019). Update of the MDS research criteria for prodromal Parkinson’s disease. Mov. Disord..

[27] Willis AW, Evanoff BA, Lian M, Criswell SR, Racette BA (2010). Geographic and ethnic variation in Parkinson disease: a population-based study of US Medicare beneficiaries. Neuroepidemiology.

[28] Moisan F (2016). Parkinson disease male-to-female ratios increase with age: French nationwide study and meta-analysis. J. Neurol. Neurosurg. Psychiatry.

[29] Gordon, N. & Lin, T. The Kaiser Permanente Northern California adult member health survey. *The Permanente J.* **20**, 15–225 (2016). 10.7812/TPP/15-225 PMC5101088 27548806

[30] Murakami K (2011). Distinct impact of education and income on habitual exercise: a cross-sectional analysis in a rural city in Japan. Soc. Sci. Med..

[31] Meltzer DO, Jena AB (2010). The economics of intense exercise. J. Health Econ..

[32] Ahlskog JE (2011). Does vigorous exercise have a neuroprotective effect in Parkinson disease?. Neurology.

[33] Cruise K (2011). Exercise and Parkinson’s: benefits for cognition and quality of life. Acta Neurologica Scandinavica.

[34] Xu Q (2010). Physical activities and future risk of Parkinson disease. Neurology.

[35] Speelman AD (2011). How might physical activity benefit patients with Parkinson disease?. Nat. Rev. Neurol..

[36] Dubé JJ, Fleishman K, Rousson V, Goodpaster BH, Amati F (2012). Exercise dose and insulin sensitivity: relevance for diabetes prevention. Med. Sci. Sports Exerc..

[37] Hu G, Lakka TA, Kilpeläinen TO, Tuomilehto J (2007). Epidemiological studies of exercise in diabetes prevention. Appl. Physiol. Nutr. Metab..

[38] Pagano G (2018). Diabetes mellitus and Parkinson disease. Neurology.

[39] Cereda E, Barichella M, Cassani E, Caccialanza R, Pezzoli G (2012). Clinical features of Parkinson disease when onset of diabetes came first: a case-control study. Neurology.

[40] Sun Y (2012). Risk of Parkinson disease onset in patients with diabetes: a 9-year population-based cohort study with age and sex stratifications. Diabetes Care.

[41] Kotagal V (2013). Diabetes is associated with postural instability and gait difficulty in Parkinson disease. Parkinsonism Relat. Disord..

[42] Yue X (2016). Risk of Parkinson disease in diabetes mellitus: an updated meta-analysis of population-based cohort studies. Med. (Baltim.).

[43] Dhiman V (2021). A systematic review and meta-analysis of prevalence of epilepsy, dementia, headache, and Parkinson disease in India. Neurol. India.

[44] Aharon-Peretz J, Rosenbaum H, Gershoni-Baruch R (2004). Mutations in the glucocerebrosidase gene and Parkinson’s disease in Ashkenazi Jews. N. Engl. J. Med..

[45] Alladi PA (2009). Absence of age-related changes in nigral dopaminergic neurons of Asian Indians: relevance to lower incidence of Parkinson’s disease. Neuroscience.

[46] Owens DK (2020). Screening for cognitive impairment in older adults: US Preventive Services Task Force Recommendation statement. Jama.

[47] Berg D (2015). MDS research criteria for prodromal Parkinson’s disease. Mov. Disord..

[48] Pagan FL (2012). Improving outcomes through early diagnosis of Parkinson’s disease. Am. J. Manag Care.

[49] Tinelli, M., Kanavos, P. & Grimaccia, F. *the Value of Early Diagnosis and Treatment in Parkinson’s Disease: A Literature Review of the Potential Clinical and Socioeconomic Impact of Targeting Unmet Needs in Parkinson’s Disease* (The London School of Economics and Political Science, 2016).

[50] Denisova IA (2020). Estimating economic efficiency of preclinical diagnostics of Parkinson disease with cost-utility approach. Popul. Econ..

[51] Cubo E (2010). Pharmacotherapy in the management of early Parkinson’s disease: cost-effectiveness and patient acceptability. Clinicoecon Outcomes Res.

[52] Plassman BL (2007). Prevalence of dementia in the United States: the aging, demographics, and memory study. Neuroepidemiology.

[53] Ebly EM, Parhad IM, Hogan DB, Fung T (1994). Prevalence and types of dementia in the very old: results from the Canadian Study of Health and Aging. Neurology.

[54] Jellinger KA, Attems J (2010). Prevalence of dementia disorders in the oldest-old: an autopsy study. Acta Neuropathologica.

[55] Aarsland D, Brønnick K, Larsen J, Tysnes O, Alves G (2009). Cognitive impairment in incident, untreated Parkinson disease: the Norwegian ParkWest study. Neurology.

[56] Botta-Orfila T (2012). Age at onset in LRRK2-associated PD is modified by SNCA variants. J. Mol. Neurosci..

[57] Lüth T (2020). Age at onset of LRRK2 p. Gly2019Ser is related to environmental and lifestyle factors. Mov. Disord..

[58] Healy DG (2008). Phenotype, genotype, and worldwide genetic penetrance of LRRK2-associated Parkinson’s disease: a case-control study. Lancet Neurol..

[59] Elbaz A (2009). Professional exposure to pesticides and Parkinson disease. Ann. Neurol..

[60] Wilk J (2006). Herbicide exposure modifies GSTP1 haplotype association to Parkinson onset age: the GenePD Study. Neurology.

[61] Taylor KSM, Cook JA, Counsell CE (2007). Heterogeneity in male to female risk for Parkinson’s disease. J. Neurol. Neurosurg. Psychiatry.

[62] Wooten G, Currie L, Bovbjerg V, Lee J, Patrie J (2004). Are men at greater risk for Parkinson’s disease than women?. J. Neurol. Neurosurg. Psychiatry.

[63] Haaxma CA (2007). Gender differences in Parkinson’s disease. J. Neurol. Neurosurg. Psychiatry.

[64] Canonico, M. et al. Increased risk of Parkinson’s disease in women after bilateral oophorectomy. *Mov. Disord.* **36**, 1696–1700 (2021). 10.1002/mds.28563 33724550

[65] Rocca WA (2008). Increased risk of parkinsonism in women who underwent oophorectomy before menopause. Neurology.

[66] Blauwendraat C (2021). Investigation of autosomal genetic sex differences in Parkinson’s disease. Ann. Neurol..

[67] Vaidya B, Dhamija K, Guru P, Sharma SS (2021). Parkinson’s disease in women: Mechanisms underlying sex differences. Eur. J. Pharm..

[68] Vlaar T (2018). Association of Parkinson’s disease with industry sectors: a French nationwide incidence study. Eur. J. Epidemiol..

[69] Cerri S, Mus L, Blandini F (2019). Parkinson’s disease in women and men: what’s the difference?. J. Parkinson’s Dis..

[70] Ou Z (2021). Global trends in the incidence, prevalence, and years lived with disability of Parkinson’s disease in 204 countries/territories from 1990 to 2019. Front. Public Health.

[71] Almallouhi E (2020). Teleneurology network to improve access to neurologists for patients in rural areas: a real-world experience. Telemed. e-Health.

[72] Rukovets O (2019). Global burden of motor neuron disease is growing, signaling need for more specialists. Neurol. Today.

[73] Majersik JJ (2021). A shortage of neurologists—we must act now: a report from the AAN 2019 Transforming Leaders Program. Neurology.

[74] Leira EC, Kaskie B, Froehler MT, Adams HP (2013). The growing shortage of vascular neurologists in the era of health reform: planning is brain!. Stroke.

[75] Kamel F (2007). Pesticide exposure and self-reported Parkinson’s disease in the agricultural health study. Am. J. Epidemiol..

[76] Ojo OO (2020). The Nigeria Parkinson disease registry: process, profile, and prospects of a collaborative project. Mov. Disord..

[77] Bhidayasiri R (2011). A national registry to determine the distribution and prevalence of Parkinson’s disease in Thailand: implications of urbanization and pesticides as risk factors for Parkinson’s disease. Neuroepidemiology.

[78] Bertoni JM, Sprenkle PM, Strickland D, Noedel N (2006). Evaluation of Parkinson’s disease in entrants on the Nebraska State Parkinson’s Disease Registry. Mov. Disord..

[79] Tiwari RC, Clegg LX, Zou Z (2006). Efficient interval estimation for age-adjusted cancer rates. Stat. Methods Med. Res..

[80] Bureau, U. S. C. *International Data Base*, https://www.census.gov/programs-surveys/international-programs/about/idb.html (2021).

[81] Mollié, A. Bayesian and empirical Bayes approaches to disease mapping. *Disease Mapping and Risk Assessment for Public Health*, 15–29 (WHO, 1999).

