# Supplementary material for: Incidence of Parkinson disease in North America
Source: NPJ Parkinsons Dis. 2022 Dec 15;8:170. doi: 10.1038/s41531-022-00410-y (PMC9755252; doi:10.1038/s41531-022-00410-y)
Supplement: Supplementary file 1 — Supplemental Table 1 [file 41531_2022_410_MOESM1_ESM.docx]

| Supplemental Table 1. Data for Figure 2 Incident Cases per 100, 000 of Parkinson Disease by study, sex, 2012. | | | |
| --- | --- | --- | --- |
| Females | | Males | |
| Ages 65-74* | Incidence (95%CI) | Ages 65-74 | Incidence (95%CI) |
| Ontario | 95 (87 – 103) | Ontario | 150 (139 – 161) |
| Medicare | 99 (94 – 104) | Medicare | 173 (165 – 180) |
| HAAS | --- | HAAS | 82 (62 – 105) |
| KNPC | 76 (59 – 96) | KNPC | 131 (107 – 158) |
| REP ^a^ | 71 (19 – 182) | REP ^a^ | 126 (46 – 274) |
|  |  |  |  |
| Ages 75-84 |  | Ages 75-84 |  |
| Ontario | 191 (177 – 206) | Ontario | 338 (317 – 360) |
| Medicare | 224 (217 – 232) | Medicare | 414 (402 – 428) |
| HAAS | --- | HAAS | 199 (161 – 240) |
| KNPC | 136 (108 – 168) | KNPC | 222 (181 – 269) |
| REP ^a^ | 86 (18 – 252) | REP ^a^ | 216 (79 – 473) |
|  |  |  |  |
|  |  |  |  |
| Ages 85+ |  | Ages 85+ |  |
| Ontario | 203 (183 – 226) | Ontario | 444 (401 – 491) |
| Medicare | 236 (226 – 247) | Medicare | 439 (415 – 463) |
| HAAS | --- | HAAS | 139 (92 – 195) |
| KNPC | 64 (37 – 102) | KNPC | 165 (107 – 244) |
| REP | ** | REP | 198 (4-717) |
| Abbreviations: HAAS= Honolulu-Asia Aging Study; KPNC= Kaiser Permanente Northern California, REP=Rochester Epidemiology Project. * Presented in 10-year increments to allow comparison to prior studies ** For this year, no females aged 85+ were diagnosed in Olmstead County, MN  ^a^ Confidence intervals between male and female estimates overlap. | | | |
